# Gender specific quality of life in patients with oral squamous cell carcinomas

**DOI:** 10.1186/1746-160X-6-21

**Published:** 2010-08-20

**Authors:** Oliver Maciejewski, Ralf Smeets, Frank Gerhards, Andreas Kolk, Frank Kloss, Jamal M Stein, Adrian Kasaj, Felix Koch, Maurice Grosjean, Dieter Riediger, Sareh Said Yekta

**Affiliations:** 1 University Hospital Aachen, Department of Oral and Maxillofacial Surgery, Aachen, Germany; 2 RWTH Aachen University, Interdisciplinary Center for Clinical Research Aachen, Aachen, Germany; 3 Technische Universität of Munich, Department of Oral and Cranio-Maxillofacial Surgery, Munich, Germany; 4 Medical University of Innsbruck, Department of Cranio-Maxillofacial and Oral Surgery, Innsbruck, Austria; 5 Faculty of Medicine, University of Mainz, Department of Oral and Maxillofacial Surgery, Mainz, Germany

## Abstract

**Background:**

The goal of this study was to evaluate the somatic and psychological effects by means of QUALITY OF LIFE (QOL) of surgical treatment of patients with oral squamous cell carcinoma. The factors gender, age, nicotine consumption, and tumour stage were taken into consideration.

**Methods:**

54 patients after surgical resection of oral squamous cell carcinomas (OSCC) were analysed from 01.09.2005 to 31.05.2008. Inclusion criteria for the study were: age at least 18 years, no indication or treatment of synchronous and metachronous tumours.

German translations of the EORTC H&N-35 and EORTC QLQ-C-30 questionnaires, as well as a general socioeconomic patient history were used as measuring instruments. The questionnaires were completed independently by the patients. The answers were translated into scale values for statistical evaluation using appropriate algorithms.

**Results:**

Analysis of the EORTC-QLQ-C-30 questionnaires demonstrated a tendency of more negative assessment of emotional function among the female participants, and a more negative evaluation of social function among the male participants. Greater tumour sizes showed significantly lower bodily function (p = 0.018). While a smaller tumour size was significantly associated with lower cognitive functioning (p = 0.031). Other cofactors such as age, nicotine consumption, and tumour stage only showed a tendency to influence the quality of sleep and daily life.

**Conclusions:**

The data obtained within this investigation demonstrated that gender had the most significant power on the subjectively perceived postoperative quality of life. This factor is important e.g. in preoperative decision making regarding immediate microvascular reconstruction after e.g. mandibular resection and therefore QOL assessment should become integral component of the care of patients with OSCC.

## Background

The treatment of head and neck malignancies involves surgical resection and adjuvant radio- and/or chemotherapy, if indicated. The main patient's concerns are survival time, and the secondary functional deficits resulting from surgery and adjuvant therapy. Therapeutic success should not only be measured on absence of recurrence and metastases, but also on the characteristics that indicate the QOL, which is defined according to the WHO (1947) as the factors in life of an individual that are important to him or her and as complete physical, mental, and social well-being. Other authors affiliate health associated QOL with the difference between the expectations of a patient, and the status that is achievable [1]. Shumaker defines QOL as a subjective evaluation in which the level of health, medical care, and supportive therapy influence the human capacity to achieve and maintain one's goals in life [2].

QOL studies give the clinician information on the effects of diseases, their treatments, and the side effects of these [2]. Patients benefit from such studies, as they can define the most disturbing aspects of the disease, and can contribute to therapy decisions, because an increase in survival time is not necessarily associated with an improvement in the QOL [3]. In previous studies, the complaints associated with disease and treatment were assessed worse by patients themselves than by the attending physicians [4,5]. Hence, a patient's assessment should be integrated in the evaluation of treatment results.

QOL studies are an established instrument in oncological research in developed countries [6-8]. However, QOL is difficult to evaluate, and can only be measured indirectly. Questionnaires on this offer the advantage that the most common complaints can be compiled in a structured manner.

The European Organization for Research and Treatment (EORT) introduced a questionnaire (Quality of Life Questionnaire 30-QLQ-C30) that evaluates general aspects of QOL associated with various tumours. This questionnaire has gained worldwide acceptance, and has been translated into many different languages [9,10]. This questionnaire contains a special section for head and neck tumours: 35-QLQ-H&N35 [11].

The number of publications concerning the measurement of QOL in patients with head and neck tumours has risen recently [8,12,13]. Typical Head and neck tumours treated by maxillofacial surgeons influence multiple functions, including respiration, food intake, speech, and socialisation through aesthetic impairment [10]. These are a heterogeneous group of tumours, although they belong to the same patho-anatomical family: oral cavity, oropharynx, larynx, hypopharynx, nasopharynx, sinus, and salivary gland tumours. The various entities influence different functions, and thus the QOL, in ways that must be distinguished. Differences between the oral cavity and oropharynx entities were first described in a study by Chandu [7].

Up to now, very few other studies differentiate between the various head and neck tumours. Previous investigations were limited to oral and oropharyngeal tumours [8,14].

In the current study, the EORTC-QLQ-C30 and its special questionnaire QLQ-H&N35 were used to evaluate a group of patients with oral malignant tumours with special focus on the influences of age, gender, nicotine consumption, and tumour stage.

## Methods

In a cross-sectional study, 73 patients with oral squamous cell carcinomas of the tongue, gingiva, buccal mucous membrane, hard and soft palates, and floor of the mouth were evaluated at one month following completion of surgical therapy in the Department of Oral and Maxillofacial Surgery at the university hospital in Aachen, Germany from 01.09.2005 to 31.05.2008. All patients received free flap reconstruction after tumorresection by the same operational team with at least one decade of experience in this operational field. One surgeon performed tumorresection and flapraising, the other surgeon always performed neck dissection and reconstruction. The study was approved by the local ethics committee, and all patients signed a written consent form.

Exclusion criteria for the study were: poor general health, serious coexisting disease, synchronous and metachronous tumours, recurrence of malignancy, and psychological or psychomotoric dysfunction that would hinder adequate completion of the questionnaire. Among the 73 patients who consented to participate in this study, 54 patients completed the QOL assessment criteria and were eligible for evaluation (Table 1). 19 Patients could not be included on the grounds that they were deceased, unable to be reached by telephone or mail, or declined to participate for personal reasons.

**Table 1:** Characteristics of all 54 patients in the study, Data are number (%) of patients

	Total n (%)	Male n (%)	Female n (%)
Age Median (SD)	60 (11)	60 (10)	61 (14)
Gender	54 (100)	31 (57)	23 (43)
			
Level of education completed			
A-levels	11 (20)	9 (17)	2 (4)
6 year secondary school	9 (17)	6 (11)	3 (6)
Basic secondary school	33 (61)	15 (28)	18 (33)
No diploma	1 (2)	1 (2)	0 (0)
			
T			
< 4 cm (T 1-2)	44 (81)	26 (48)	18 (33)
> 4 cm (T 3-4)	10 (19)	5 (9)	5 (9)
			
Alcohol consumption			
Never	5 (9)	2 (4)	3 (6)
Seldom	43 (80)	26 (48)	17 (31)
Regularly	6 (11)	3 (6)	3 (6)
			
Nicotine consumption			
Yes	29 (54)	16 (30)	13 (24)
No	25 (46)	15 (28)	10 (19)

The patient data were gathered with a socioeconomic questionnaire containing data about age, gender, marital status, level of education, occupation, coexisting diseases, nicotine and alcohol consumption, and tumour stage and localisation at the time of diagnosis. QOL was evaluated using the EORTC-QLQ-C30 Version 3.0 and its supplement H&N35, in German translation [15].

The patients completed the questionnaire in the presence of an expert, who was able to assist in case of difficulties in understanding the questions, but did not influence the answers. The EORTC-QLQ-C30 questionnaire is concerned with malignant diseases and their treatment in general, and contains 30 questions. These questions are divided into the following topics: global functioning (mobility, ability to work, emotional stress, cognitive stress, and social stress), general symptoms and problems (fatigue, nausea and vomiting, pain, dyspnea, sleep disturbances, lack of appetite, constipation, diarrhea, and financial burden) and general level of health/quality of life. The answer possibilities for the topic "general health and quality of life" encompass a scale from 1 - 7, where 1 represents the appraisal "very poor", and 7 "excellent". The topics "global functioning" and "general symptoms and problems" have the answer alternatives 1 (not at all), 2 (somewhat), 3 (moderate), and 4 (extreme). The answers to the individual questions are represented on a scale of 0 - 100. A high score on the scale represents severe problems [16].

The EORTC-H&N-35 describes the disease specific QOL in the head and neck malignancies. The questionnaire is a supplement to the QLQ-C30, and contains 35 questions. There are 7 scaled answers for pain, swallowing, sensibility, speech, eating in a social setting, social contact, and sexuality. Further, 11 individual topics are evaluated taking localisation, symptoms, and treatment into account (dental problems, mouth opening, dry mouth, poor salivation, cough, sense of illness, analgesic use, nutrition difficulties, gastric tube, weight loss or gain). Analogous to the QLQ-C30, the answers are represented on a scale of 0-100, and a high score on the scale of symptoms describes severe problems [16]. The scores of each of these questionnaires were interpreted according to the EORTC guidelines. The statistical analysis of the data was completed using SPSS1 version 14.0 (SPSS Inc.). A p-value < 0.05 was considered as statistically significant. The descriptive statistics were conducted using the absolute and relative frequencies of the qualitative variables. The distribution of quantitative variables was determined using the mean and standard deviation (determined as normal or abnormal using the Kolmogorov-Smirnov test). The Mann-Whitney U - or the Kruskall-Wallis tests were used to compare various groups of quantitative variables, as these demonstrated an abnormal distribution. Qualitative variables were analysed using the Chi^2 ^test or Fisher's exact test.

## Results

Tables 2, 3, 4, and 5 show the distribution of medians and interquartile ranges of the scales function, symptoms, and for general health and quality of life for the EORTC-QLQ-C30 questionnaire, comparing the patient groups according to gender, nicotine consumption, age (< 60 years and > 60 years), and tumour stage.

**Table 2:** EORTC QLQ-C30 Gender

	male P50 [P25-P75] n (31)	female P50 [P25-P75] n (23)	P value
General health	33 [16-50]	33 [16-50]	0.837
Bodily function	75 [47-86]	73 [47-80]	0.979
Role functioning	33 [0-83]	33 [0-67]	0.928
Emotional functioning	50 [33-75]	67 [50-75]	0.340
Cognitive functioning	67 [0-83]	67 [0-83]	0.484
Social functioning	50 [0-67]	16 [0-67]	0.789
			
Fatigue	33 [10-67]	33 [0-67]	0.676
Nausea/Vomiting	0 [0-33]	0 [0-33]	0.844
Pain	33 [33-67]	33[0-67]	0.606
Dyspnea	33[30-33]	0 [0-66]	0.483
Sleep disorders	66 [0-100]	33 [33-87]	0.415
Loss of appetite	33 [0-67]	0 [0-67]	0.288
Constipation	0 [0-33]	0 [0-100]	0.592
Diarrhea	0 [0-33]	0 [0-33]	0.711
Financial stress	33 [0-67]	0 [0-67]	0.770

**Table 3:** EORTC QLQ-C30 Nicotine consumption

	Smoker P50 [P25-P75] n (29)	Non-smoker P50 [P25-P75] n (25)	P value
General health	33 [16-50]	33 [0-41,5]	0.116
Bodily function	75 [53.5-86]	73 [43.5-80]	0.589
Role functioning	50 [0-91.5]	33 0-67]	0.327
Eotional functioning	67 [33-74]	58 [16.5-75]	0.495
Cognitive functioning	67 [0-83]	67 [0-83]	0.978
Social functioning	50 [0-75]	16 [0-67]	0.416
			0.937
Fatigue	33 [5-67]	33 [5,5-67]	0.639
Nausea/Vomiting	0 [0-33]	0 [0-33]	0.244
Pain	33[0-67]	33 [33-67]	0.953
Dyspnea	16.5 [0-66]	33 [0-33]	0.389
Sleep disorders	66 [33-100]	33 [0-100]	0.135
Loss of appetite	0 [0-33]	33 [0-100]	0.827
Constipation	0 [0-83.5]	0 [0-67]	0.730
Diarrhea	0 [0-33]	0 [0-33]	0.654
Financial stress	0 [0-67]	33 [0-67]	0.116

**Table 4:** EORTC QLQ-C30 T- Stage

	T1 P50 [P25-P75] n [34]	T2 P50 [P25-P75] n [10]	T3 P50 [P25-P75] n [3]	T4 P50 [P25-P75] n [7]	p[T1/T2-T3/T4]
General health	33 [12-50]	29 [12-37.5]	33 [16-50]	33 [25-33]	> 0.999
Bodily function	75 [53-81.5]	83 [58.5-86.25]	40 [13-73]	67 [27-75]	0.018
Role functioning	16.5 [0-67]	58.5 [12-100]	33 [17-100]	50 [0-67]	0.432
Emotional functioning	58 [0-77]	74 [45.75-79.25]	75 [67-92]	33 [33-67]	0.719
Cognitive functioning	25 [0-75]	71 [0-83]	67 [0-100]	83 [67-83]	0.031
Social functioning	16 [0-67]	67 [0-87.25]	50 [0-67]	50 [0-67]	0.752
					
Fatigue	27.5 [0-58.75]	61.5 [24.75-67]	100 [22-100]	67 [0-100]	0.098
Nausea/Vomiting	0 [0-20.25]	8 [0-37.25]	50 [33-100]	0 [0-16]	0.187
Pain	33 [0-67]	58.5 [0-71]	33 [33-83]	33 [0-83]	0.800
Dyspnea	33 [0-66]	33 [0-50]	33 [0-33]	16.5 [0-49.75]	0.846
Sleep disorders	66 [24.75-100]	50 [25.75-100]	33 [0-33]	33 [0-100]	0.218
Loss of appetite	0 [0-41.4]	33 [0-67]	100 [0-100]	0 [0-67]	0.475
Constipation	0 [0-100]	0 [0-0]	0 [0-33]	0 [0-67]	0.630
Diarrhea	0 [0-33]	0 [0-33]	0 [0-100]	0 [0-33]	0.438
Financial stress	0 [0-67]	33 [-75.25]	0 [0-67]	33 [0-100]	0.614

**Table 5:** EORTC QLQ-C30 Age

	Age <60 P50 [P25-P75] n [27]	Age >60 P50 [P25-P75] n [27]	P value
General health	33 [16-50]	33 [16-50]	0.784
Bodily function	75 [33-86]	73 [53-80]	0.917
Role functioning	50 [0-67]	17 [0-83]	0.495
Emotional functioning	67 [33-75]	58 [0-75]	0.553
Cognitive functioning	67 [0-83]	67 [0-83]	0.679
Social functioning	67 [0-83]	0 [0-67]	0.051
			
Fatigue	55 [33-67]	22 [0-67]	0.083
Nausea/Vomiting	0 [0-16]	0 [0-33]	0.520
Pain	50 [0-83]	33 [0-50]	0.093
Dyspnea	33 [0-66]	16.5 [0-33]	0.537
Sleep disorders	33 [33-100]	33 [0-100]	0.761
Loss of appetite	0 [0-67]	0 [0-33]	0.379
Constipation	0 [0-67]	0 [0-33]	0.925
Diarrhea	0 [0-33]	0 [0-0]	0.097
Financial stress	33 [0-100]	0 [0-67]	0.361

The gender dependent analysis of quality of life obtains the trend that emotional functioning, composed of the factors tension, worry, irritability, and depression was judged to be worse by the females (Table 2). In contrast, the male group demonstrated higher scores (poorer function) for social functioning, which incorporated familial and general relations with other persons. The male participants tended to rate worse on the functional and environmental symptom scales for dyspnea, sleep disorders, and financial stress. A comparison of the genders in relation to the tumour specific symptoms indicated that the females tended to show more severe symptoms in swallowing, salivation, and coughing as well as weight loss (5 kg for females vs. 2 kg for males).

Smokers generally tended to score worse than non-smokers in emotional, social, cognitive, and role functioning and revealed more sleep dysfunction (Table 3). Non-smokers judged the symptoms of dyspnea and financial stress more negatively than the latter. The tumour specific symptoms speech, swallowing, social contact, and dental health were worse in the smoker's group. The non-smokers had more complaints of dry mouth and cough. The smokers lost, with a median of 4 kg, more weight in comparison to the non-smokers with a median of 2 kg.

The degree of discrimination for ascertaining the significance levels was determined for a tumour size of 4 cm, with the stages T1/2 versus T3/4. The greater tumour sizes T3/4 exhibited a significantly lower bodily function (p = 0.018; Table 4). Emotional functioning was given the worst assessment by participants in stage T4. While cognitive and social functioning was rated to be very high by patients with a tumour size of at least 2 cm (T2-T4), it was found to be statistically significant that a smaller tumour size was associated with lower cognitive functioning (p = 0.031). On the symptom scales, a smaller tumour size (T1/2) was associated with more sleep disorders. No clear tendencies could be found comparing the tumour specific symptom scales for the various tumour stages, however speech and social contact tended to show the worst values for the stages T1/2. Weight loss increased with greater tumour size (median values: T1 = 2 kg, T2 = 5.5 kg, T3 und T4 = 7kg).

In relation to QOL, solely social functioning showed the tendency to be age-related as it was determined to be better for patients under 60 years (p = 0.051; Table 5).

## Discussion

OSCC and its treatment directly affect health-related QOL. The most basic functions of speech, chewing and swallowing are frequently altered, while symptoms such as pain and psychosocial issues like appearance and emotional functioning can also be problematical.

Most studies that are concerned with QOL for head and neck tumours do not differentiate between the subgroups of various tumour localisations. Some investigators criticise the heterogeneity of these studies, since large differences in the assessment of QOL could be found between the individual localisations with the questionnaires [17]. Other authors have negated these differences [18].

At present, there is no universally accepted QOL questionnaire for patients with OSCC, which results in difficulties when attempting to compare the outcome of different institutions [19]. Therefore the established EORTC-QLQ-C30/H&N-35 and the questionnaires were used in this study. In a comparison of different questionnaires like the University of Washington (UW) Head and Neck Disease-Specific Measure, the Medical Short Form 36 and the EORTC-H&N35, the latter was more sensitive in detecting changes in the single items of speech and swallowing, and furthermore the UW-QOL does not explore emotional, cognitive and social function [20]. Other authors found that the UW-QOL scale is most suitable for surgical patients [21]. Our experience has confirmed that the 65 items of the EORTC-QLQ-C30/H&N-35 cover most important issues of patients receiving treatment for head and neck cancer and provides a reasonable assessment. Two studies have been published that measured QOL at time of diagnosis using the EORTC-QLQ-C30/H&N35. In the Netherlands a study with 80 patients demonstrated QOL before therapy for oral and oropharyngeal tumours correlated with tumour localisation, stage, and comorbidity [6]. Patients with oral cavity (mobile tongue, gums, floor of the mouth, buccal mucosa, hard palate and buccal area of the soft palate) tumours reported more pain than patients with oropharyngeal (located behind the anterior pillar of the pharynx, retromolar trigone, tonsils, tonsillar region of the soft palate and base of tongue) tumours. Patients with advanced stage tumours (T3/4) showed more obstruction to mouth opening and a higher sense of illness than patients with T1/2 tumours.

In a multi-centre study of 357 patients with head and neck tumours in Sweden and Norway, differences in quality of life were determined for tumour localisation (patients with oral tumours reported more pain), advanced tumour stage, gender (females scored more poorly on the emotional scale), and age (patients over 65 years showed better scores on the emotional and social scales) [22].

The present cross-sectional study shows that the scales for general health/quality of life, role and social functioning were negatively influenced, in contrast to the scales for bodily, emotional functioning and cognitive functioning, which tended to be rated more positively.

With regard to symptom assessment, fatigue, pain, dyspnoea, sleep disorders, and financial stress were rated more negatively than the symptoms nausea and vomiting, lack of appetite, constipation, and diarrhea.

Some studies did not observe any differences between the genders [20]. In our study, females tended to show more negative scores in most of the function subgroups, especially for emotional functioning, which consisted of the factors tension, worry, irritability, and depression. Furthermore, the female gender also demonstrated worse ratings for swallowing, salivation, and coughing. In contrast, the male group rated social functioning more negatively, which encompassed the areas of familial and general relations with other persons. The males tended to score dyspnea, sleep disorders, and financial stress more negatively, which resulted in a higher level of psychological stress as compared to the female gender. The results of another study were contrary [22]. Bjordal showed that QOL assessment was lower for females, but these values equalised after one year, when more mental changes, alcohol problems, and poor nutrition were found among the males [10].

The gender specific results of the present study also correspond with age, since tumour diagnoses among females were made at an older age, and mostly at an earlier stage than in males. The correlation between age and many of the QOL subgroups, such as the bodily symptoms of dry mouth and dental problems can be explained by natural physical decline in advanced age [8]. The social and emotional subgroups are two exceptions, since the assessment of these by younger patients is normally more negative than by older patients [23]. Contrary to this, the present study showed a higher evaluation of social function by patients less than 60 years.

In the present study, smokers tended to demonstrate a lower evaluation of social, emotional, cognitive, and role functioning than non-smokers. For non-smokers, the symptoms of dyspnea and financial stress were judged to be worse than by smokers. The smokers however exhibited greater sleep dysfunction, and a more negative impact on speech, swallowing, loss of social contact, and dental problems, some of which could be explained by withdrawal symptoms. Complaints of dry mouth and cough were increased among non-smokers, symptoms that can be triggered by nicotine consumption.

Posterior localised tumours demonstrate a worse prognosis, since these often remain unnoticed in screening examinations, and once symptoms arise from regional lymph node metastases, the tumours are at an advanced stage at time of initial diagnosis [24,25].

At the time of diagnosis, the non-specific symptoms of oral tumours include fatigue, nausea, vomiting, and loss of appetite. Fatigue and loss of appetite can be explained by a decline in the general state of health through an advanced stage tumour. Oral tumours, especially those in the posterior region can stimulate the emetic impulse, and can obstruct the passage of a bolus during swallowing, and induce nausea and vomiting in this manner.

Despite the fact, that in the present study all operations were performed by the same team, the surgeons dexterity always biases surgical related investigations. To our knowledge no study exists so far, which has been able to eliminate this bias.

## Conclusions

Prospective QOL assessment can provide valuable additional information for both the treatment team and the patients. In addition, it gives an opportunity to support routine medical follow up. The present study included only patients with oral tumours from the heterogenous group of head and neck malignancies. Hence, the number of patients was limited, which influenced non-significant results, and might explain the contrary nature of the results, in comparison to the existing literature. Even oral tumours are heterogenous, and tumours in the anterior region of the floor of the mouth show different symptoms than posterior tumours, or malignancies in the buccal or palatal regions. With regard to QOL studies, a more specific differentiation in this area is desirable. Consequently, further prospective studies must explore this topic with larger patient collectives.

## Competing interests

The authors disclose any financial and personal relationships with other people or organisations that could inappropriately influence their work.

## Authors' contributions

OLM participated in the design and coordination of the study and helped to draft the manuscript, FG helped to draft the manuscript, RS and SSY participated in the design and coordination of the study and helped to draft the manuscript. All authors read and approved the final manuscript.

## References

[B1] Dietz A Meyer A Singer S Lebensqualitätsmessungen bei Patienten mit Kopf-Hals-Malignomen. Aktueller Stand und zukünftige Anforderungen HNO 2009 57 857 865 10.1007/s00106-009-1969-1 19629416

[B2] Shumaker S Naughton M Berzon R, Shumaker S The international assessment of health related quality of life: a theoretical perspective The International Assessment of Health-Related Quality of Life: Theory, Translation, Measurement and Analysis 1995 Oxford, Rapid Communications of Oxford 3 10

[B3] Rogers SN Ahad SA Murphy AP A structured review and theme analysis of papers published on 'quality of life' in head and neck cancer: 2000-2005 Oral Oncol 2007 43 843 868 10.1016/j.oraloncology.2007.02.006 17600755

[B4] Kahn SB Hums PS Harding SP Quality of life and patients with cancer: A comparative study of patients versus physicians perceptions and its implications for cancer education J Cancer Ed 1992 7 421 10.1080/08858199209528175 1419591

[B5] Presant CA Quality of life in cancer patients: Who measures what? Am J Clin Oncol 1984 7 571 10.1097/00000421-198410000-00036 6507381

[B6] Borggreven PA Verdonck-De Leeuw IM Muller MJ Heiligers ML De Bree R Aaronson NK Leemans CR Quality of life and functional status in patients with cancer of the oral cavity and oropharynx: pretreatment values of a prospective study Eur Arch Otorhinolaryngol 2007 264 651 657 10.1007/s00405-007-0249-5 17273840 PMC1914238

[B7] Chandu A Smith AC Rogers SN Health-related quality of life in oral cancer: a review J Oral Maxillofac Surg 2006 64 495 502 10.1016/j.joms.2005.11.028 16487814

[B8] Infante-Cossio P Torres-Carranza E Cayuela A Gutierrez-Perez JL Gili-Miner M Quality of life in patients with oral and oropharyngeal cancer Int J Oral Maxillofac Surg 2009 38 250 255 10.1016/j.ijom.2008.12.001 19135864

[B9] Aaronson NK Ahmedzai S Bergman B Bullinger M Cull A Duez NJ Filiberti A Flechtner H Fleishman SB de Haes JC The European Organisation for research and treatment of cancer QLQ-C30: A quality-of-life instrument for use in international clinical trials in oncology J Natl Cancer Inst 1993 85 365 376 10.1093/jnci/85.5.365 8433390

[B10] Bjordal K Hammerlid E Ahlner-Elmqvist M de Graeff A Boysen M Evensen JF Biörklund A de Leeuw JR Fayers PM Jannert M Westin T Kaasa S Quality in head and neck cancer patients: validation of the European Organisation for Research and Treatment of Cancer Quality of Life questionnaire-H&N35 J Clin Oncol 1999 17 1008 1019 10071296 10.1200/JCO.1999.17.3.1008

[B11] Bjordal K de Graeff A Fayers PM Hammerlid E van Pottelsberghe C Curran D Ahlner-Elmqvist M Maher EJ Meyza JW Brédart A Söderholm AL Arraras JJ Feine JS Abendstein H Morton RP Pignon T Huguenin P Bottomly A Kaasa S A 12 country field study of the EORTC QLQ-C30 (version 3.0) and the head and neck cancer specific module (EORTC QLQ-H&N35) in head and neck patients. EORTC Quality of Life Group Eur J Cancer 2000 36 1796 1807 10.1016/S0959-8049(00)00186-6 10974628

[B12] Mehanna HM Morton RP Deterioration in quality-of-life of late (10-year) survivors of head and neck cancer Clin Otolaryngol 2006 31 204 211 10.1111/j.1749-4486.2006.01188.x 16759240

[B13] Oates J Clark JR Read J Reeves N Gao K O'Brien CJ Integration of prospective quality of life and nutritional assessment as routine components of multidisciplinary care of patients with head and neck cancer ANZ J Surg 2008 78 34 41 10.1111/j.1445-2197.2007.04353.x 18199203

[B14] Baumann I Seibolt M Zalaman I Dietz K Maassen M Plinkert P Quality of life in patients with oropharyngeal carcinoma after primary surgery and postoperative irradiation J Otolaryngol 2006 35 332 337 10.2310/7070.2006.0077 17049151

[B15] European Organization for Research and Treatment of Cancer http://www.eortc.be/

[B16] Fayers PM Machin D Quality of Life: Assessment, Analysis and Interpretation 2007 Chichester, John Wiley and Sons

[B17] De Graeff A De Leeuw JR Ros WJ Hordijk GJ Winnubst JA Pretreatment factors predicting quality of life after treatment for head and neck cancer Head Neck 2000 22 398 407 10.1002/1097-0347(200007)22:4<398::AID-HED14>3.0.CO;2-V 10862025

[B18] Morton RP Izzard ME Quality of Life Outcomes in Head and Neck Cancer Patients World J Surg 2003 27 884 889 10.1007/s00268-003-7117-2 14509523

[B19] Murphy BA Ridner S Wells N Dietrich M Quality of life research in head and neck cancer: A review of the current state of the science Critical Reviews in Oncology/Hematology 2007 62 251 267 10.1016/j.critrevonc.2006.07.005 17408963

[B20] Rogers SN Lowe D Brown JS Vaughan ED A comparison between the University of Washington Head and Neck Disease-Specific measure and the Medical Short Form 36, EORTC QOQ-C33 and EORTC Head and Neck 35 Oral Oncol 1998 34 361 372 10.1016/S1368-8375(98)00031-1 9861341

[B21] Ringash J Bezjak A A structured review of quality of life instruments for head and neck cancer patients Head Neck 2001 23 201 213 10.1002/1097-0347(200103)23:3<201::AID-HED1019>3.0.CO;2-M 11428450

[B22] Hammerlid E Bjordal K Ahlner-Elmqvist M Boysen M Evensen JF Biörklund A Jannert M Kaasa S Sullivan M Westin T A prospective study of quality of life in head and neck cancer patients. Part I at diagnosis Laryngoscope 2001 111 669 680 10.1097/00005537-200104000-00021 11359139

[B23] Coates A Porzolt F Osoba D Quality of life in oncology practise: prognostic values of EORTC QLQ C30 scores in patients with advanced malignancies Eur J Cancer 1997 33 1025 1030 10.1016/S0959-8049(97)00049-X 9376182

[B24] Rogers SN Lowe D Patel M Brown JS Vaughan ED Clinical function after primary surgery for oral and oropharyngeal cancer: an 11-item examination Br J Oral Maxillofac Surg 2002 40 1 10 10.1054/bjom.2001.0701 11883962

[B25] Infante-Cossio P Torres-Carranza E Cayuela A Hens-Aumente E Pastor-Gaitan P Gutierrez-Perez JL Impact of treatment on quality of life for oral and oropharyngeal carcinoma Int J Oral Maxillofac Surg 2009 38 1052 1058 10.1016/j.ijom.2009.06.008 19596557

